# In search of the relevant COVID research

**DOI:** 10.1093/eurpub/ckaa169

**Published:** 2020-08-20

**Authors:** Peter Allebeck, Alma Sörberg Wallin

**Affiliations:** Department of Global Public Health, Karolinska Institutet, SE-17177 Stockholm, Sweden

‘Are we publishing what we should be publishing?’ is a question all editors ask themselves from time to time. With an acceptance rate of ∼20% for the *European Journal of Public Health* (*EJPH*), and even lower for many others, one question is how we prioritize among incoming papers. But, the problem raised by Odone *et al.*^1^ is that scientists seem to have failed to address the important issues regarding the COVID-19 pandemic. Some reasons are obvious: this is a new virus with unknown properties, global spread is of a character previously unknown, case definition and cause of death assessment vary strongly making comparisons difficult, and the long-term effects are too early to evaluate. And on the positive side, we should acknowledge the extremely rapid publication of the first characterization of the disease,^2^ and the genome sequence,^3^ compared to the long road to knowledge on HIV and SARS. But, as Horton^4^ formulates it in heading of a recent book on the topic ‘*Science: the Paradox of Success and Failure*’.

Behind the increase in published papers reported by Odone *et al.*^1^ is an even stronger increase of incoming manuscripts to many scientific journals. During the pandemic, this journal has had an exceptional inflow of manuscripts. During the period February–July 2020 we received 907 manuscripts, compared with 614 manuscripts the same period 2019. The 907 manuscripts are close to the average of ∼1000 manuscripts that we receive during 1 year. Of all manuscripts submitted during February–July this year, 238 had ‘COVID’, ‘corona’ or ‘pandemic’ in the title, i.e. 26%, and there might have been more, not reflected in the title.

The great majority of these came from China, others from Italy, Iran, Turkey and some other countries. Most were empirical studies of the type found in newspapers or national public health reports on the web: regional surveys, case series, clinical outcome studies, simple comparisons from official sources and examples of new rapid hospital constructions. We were disappointed to receive so many manuscripts on a major public health issue, but with so little findings of international public health relevance, and so little new science. Almost all these papers were rejected, and we formulated a standard letter explaining that findings might be interesting, but more long term and public health relevant research is needed: ‘We need to await evaluations and see the long-term perspective in order (for the journal) to be an appropriate forum for reporting and debate. But that time will definitely come, and the *EJPH* will strongly welcome contributions to inform policy and decision making in rapid infection spread.’

So, how come so many articles were submitted and also published while in many cases not addressing questions of major scientific or public health relevance? One impression one gets after having assessed the hundreds of COVID-19-related manuscripts submitted to the *EJPH*, is that suddenly lots of data were available, and many sensed an urge to publish. Clinical data, testing reports, surveys on behavior and people’s perceptions, reformed health care facilities. Lots of things to write about, as has been done daily in national newspapers and national public health agencies’ webpages. Part of the explanation is thus the classical problem with pressure to publish. Many universities’ incentive systems are still based on quantity rather than quality and innovative strength of publications.

Odone *et al.*^1^ cite lists of priorities for COVID-19-related research. As indicated in our standard letter mentioned above, our priorities would rather focus on long-term effects and evaluation of measures taken. Here are some examples of research questions we hope to see papers on:

- What are the long-term consequences of the pandemic, taking into account economic downturn and unemployment?
- Which control measures have been more or less successful to mitigate the spread?
- To what extent have ‘lockdowns’ had an overall positive effect in the long term, or have the negative effects predominated?
- How valid are the reported national data on cases and deaths, and are there comparative studies with highly standardized data collection?

There are many other relevant research questions, but the important message is that the research should start from relevant research questions, not from available data.

*Conflicts of interest*: None declared. 
